# Structures of FOX-4 Cephamycinase in Complex with Transition-State Analog Inhibitors

**DOI:** 10.3390/biom10050671

**Published:** 2020-04-27

**Authors:** Scott T. Lefurgy, Emilia Caselli, Magdalena A. Taracila, Vladimir N. Malashkevich, Beena Biju, Krisztina M. Papp-Wallace, Jeffrey B. Bonanno, Fabio Prati, Steven C. Almo, Robert A. Bonomo

**Affiliations:** 1 Department of Chemistry, Hofstra University, Hempstead, NY 11549, USA; 2 Department of Life Sciences, University of Modena and Reggio Emilia, 41125 Modena, Italy; 3 Department of Medicine, Case Western Reserve University School of Medicine, Cleveland, OH 44106, USA; 4 Louis Stokes Cleveland Department of Veterans Affairs Medical Center, Cleveland, OH 44106, USA; 5 Department of Biochemistry, Albert Einstein College of Medicine, Bronx, NY 10461, USA; 6 Department of Biochemistry, Case Western Reserve University School of Medicine, Cleveland, OH 44106, USA; 7 Department of Pharmacology, Case Western Reserve University School of Medicine, Cleveland, OH 44106, USA; 8 Department of Molecular Biology and Microbiology, Case Western Reserve University School of Medicine, Cleveland, OH 44106, USA; 9 Department of Proteomics and Bioinformatics, Case Western Reserve University School of Medicine, Cleveland, OH 44106, USA; 10 CWRU-Cleveland VAMC Center for Antimicrobial Resistance and Epidemiology (Case VA CARES) Cleveland, OH 44106, USA

**Keywords:** β-lactam, β-lactamase, cephamycinase, boronic acid, transition-state analog inhibitor

## Abstract

Boronic acid transition-state analog inhibitors (BATSIs) are partners with β-lactam antibiotics for the treatment of complex bacterial infections. Herein, microbiological, biochemical, and structural findings on four BATSIs with the FOX-4 cephamycinase, a class C β-lactamase that rapidly hydrolyzes cefoxitin, are revealed. FOX-4 is an extended-spectrum class C cephalosporinase that demonstrates conformational flexibility when complexed with certain ligands. Like other β-lactamases of this class, studies on FOX-4 reveal important insights into structure–activity relationships. We show that SM23, a BATSI, shows both remarkable flexibility and affinity, binding similarly to other β-lactamases, yet retaining an IC_50_ value < 0.1 μM. Our analyses open up new opportunities for the design of novel transition-state analogs of class C enzymes.

## 1. Introduction

β-lactam resistance continues to increase, threatening public health worldwide. Of particular concern are carbapenem-resistant Enterobacteriaceae (CRE) that have evolved to inactivate our “last-line” therapy [1]. Dual-target strategies, in which one drug is a β-lactamase inhibitor (BLI) while a second drug (a β-lactam) inhibits the cell wall-building machinery, are increasingly used to treat infections caused by β-lactam-resistant bacteria [2]. This approach increases the usefulness of antibiotics that are otherwise susceptible to hydrolysis by β-lactamases.

**Figure 1:**
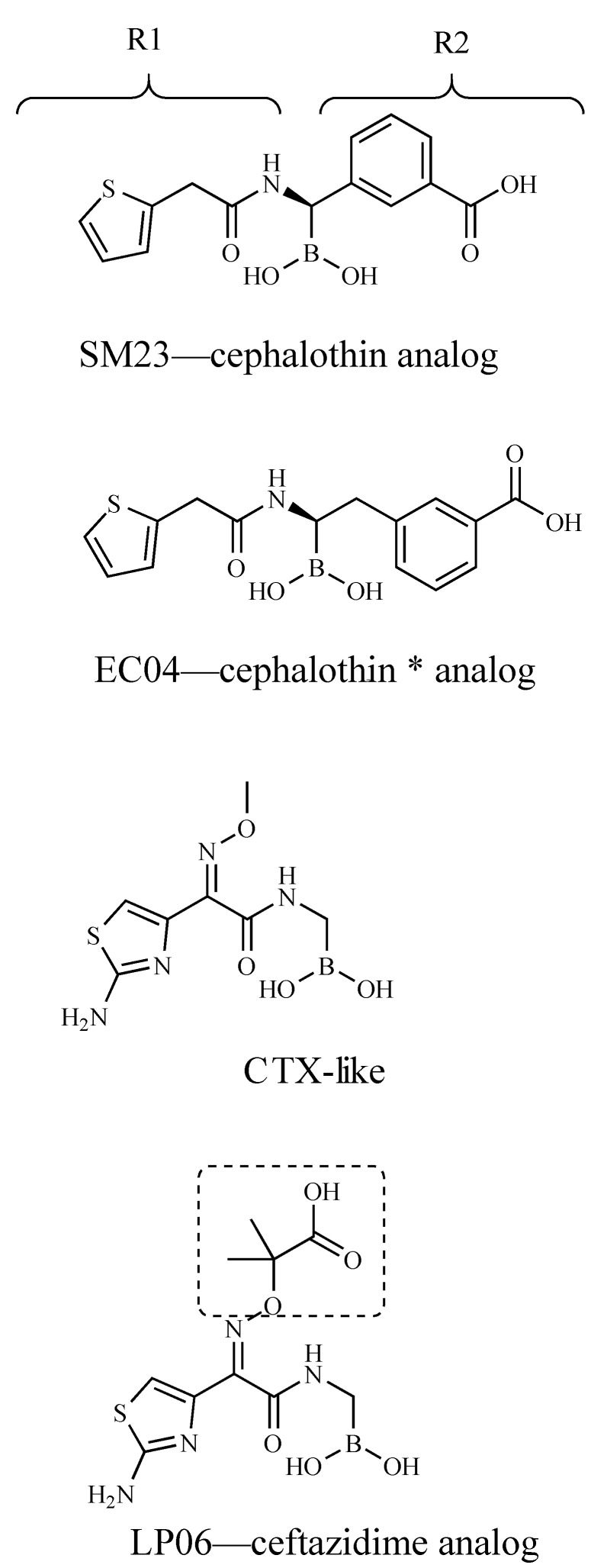
Compounds used in this study. R1 and R2 indicate side chains that are analogous to those of β-lactam substrates. Dashed lines indicate the portion of LP06 that is missing in the electron density of the FOX-4/LP06 crystal structure (PDB: 5CHM). * An extra carbon linker is present on the R2 side chain.

Boronic acid transition-state analog inhibitors (BATSIs) have emerged as promising non-β-lactam BLIs that can inhibit all four structural classes of β-lactamase [3]. In combination with existing β-lactams, BATSIs have increased the potency and efficacy of other antibiotics and were shown to have in vivo activity [4]. The first generation of BATSIs inactivated serine β-lactamases (Ambler classes A, C, and D) through a dative and reversible covalent bond to the active site serine nucleophile [5,6,7,8]. Many of these compounds contained side chains that mimicked known cephalopsorins, such as those considered in this study (Figure 1). Medicinal chemistry efforts have refined many additional side chains to great effect [9,10], including some that inhibit both serine- and metallo-β-lactamases (Ambler class B) [11]. The recent development of cyclic BATSIs, in which the substituent binds twice to the boron, has added increased potency and range to this group of BLIs [12,13,14]. Vaborbactam, a monocyclic BATSI, recently received fast-track status by the Food and Drug Administration as a BLI to be used in combination with an existing β-lactam, meropenem [15,16,17]. Vaborbactam only binds strongly to class A and C β-lactamases; several bicyclic boronates currently in development have broader inhibition profiles. A bicyclic BATSI, QPX7728, was recently reported to bind 4- to 10,000-fold more tightly to serine β-lactamases and also to metallo-β-lactamases with submicromolar affinity [18,19]. Taniborbactam (VNRX-5133), another bicyclic BATSI, inhibits all classes of β-lactamases and shows particularly high affinity to VIM family metallo-β-lactamases [20].

Inhibition strategies are especially relevant for class C enzymes, which are considered resistant to inhibition by three of the six approved BLIs (the recently approved avibactam, relebactam, and vaborbactam are the exceptions, for now). Structural studies show that BATSI binding mimics many aspects of the β-lactam interactions with AmpC [5,21]. As a result, BATSIs can also be used as structural probes of these β-lactamases. BATSI-bound crystal structures for class A β-lactamases are well established, including several CTX-M family members [10], TEM-1, SHV-1, and (importantly) KPC-2 [22,23,24]. Class C enzymes have not received the same attention—most structures are with a single enzyme, *Escherichia coli* AmpC [6,9,25,26,27], although three recent analyses describing ADC-7/BATSI and PDC/BATSI complexes have expanded this repertoire considerably [14,28,29]. Other structures include *Enterobacter cloacae* P99 and 908R.

The FOX-4 cephalosporinase–BATSI structures presented herein add a cephamycinase to this growing list. Plasmid-based extended-spectrum AmpC enzymes, such as the FOX β-lactamases, are increasingly found in clinical isolates from antibiotic-resistant infections [30,31]. FOX-4 is of interest because it rapidly hydrolyzes cephamycins [32]. These compounds contain a 7α-methoxy substituent on the cephem ring that mimics the 6α-(2)-hydroxyethyl group characteristic of carbapenems (imipenem), raising the concern that cephamycinases may evolve to hydrolyze carbapenems [33]. In addition, the FOX-4 β-lactamase shows significant ligand-induced conformational changes in the R2 loop and H10 helix.

SM23, a chiral cephalothin (CEF)-based BATSI, bears an R1 side chain with a thiophene ring and an R2 side chain with a *m*-carboxyphenyl functionality (Figure 1). This designed compound is among the most potent BATSIs, inhibiting class C β-lactamases with < 0.1 µM IC_50_ values [6]. In PDC-3, this compound is an effective inhibitor, as are a series of novel BATSI compounds [34].

In this analysis, the structure of FOX-4 in complex with SM23 and LP06, a ceftazidime mimic, is described. These structures reveal similar binding modes across distantly related class C enzymes such as ADC-7, PDC-3 and *E*. *coli* AmpC, and suggest a common mode of action. The recent ADC-7 and PDC-3 structures are now joined by the FOX-4 structures as representatives of class C β-lactamases bound to transition-state analogues. Herein, we describe these structures and compare the conformations of the inhibitors bound to FOX-4 and *E*. *coli* AmpC to each other and that of a cephamycin, cefoxitin.

## 2. Materials and Methods

Reagents and compounds. Lysogeny broth (LB) powder was purchased from ThermoFisher Scientific (Springfield, NJ, USA). Nitrocefin (NCF) was obtained from TOKU-E (Bellingham, WA, USA). Unless otherwise specified, all chemicals were from Sigma-Aldrich (St. Louis, MO, USA) and were of the highest grade available.

Minimum Inhibitory Concentrations (MICs). An *E. coli* 42015 clinical strain producing *bla*_FOX-4_ and *E. coli* TG1 carrying pBGS18^−^ *bla*_FOX-4_ were subjected to susceptibility testing; these strains were previously described [32,35]. MICs were determined using cation-adjusted Mueller–Hinton (MH) broth microdilution according to Clinical and Laboratory Standards Institute (CLSI) guidelines [36]. The ceftazidime (CAZ) concentration was varied, while a constant concentration of 10 μg/mL of the BATSI compounds was used.

Protein expression and purification. Native FOX-4 was produced as previously described [33]. Briefly, periplasmic fractions of *E. coli* expressing FOX-4 from a clinically derived plasmid were incubated with a *m*-aminophenylboronic acid resin equilibrated in a buffer containing 25 mM Tris pH 7.0 and 500 mM sodium chloride; the resin (3 mL) was transferred to a column (1 cm diameter) and the flow through was collected; the column was washed with 10 volumes of equilibration buffer (0.3 mL/min) and eluted with a buffer containing 500 mM sodium borate pH 7.0 and 500 mM sodium chloride. Fractions of 95% purity were dialyzed for kinetics (50 mM Tris pH 7.5) and concentrated for crystallization.

Steady-state kinetics. Steady-state kinetics were carried out on a Cary300 (Agilent, Palo Alto, CA, USA) UV–VIS spectrophotometer as described previously [34]. Briefly, the reporter substrate, NCF, and inhibitors were dissolved in 10 mM phosphate-buffered saline pH 7.4 (PBS). β-lactamase stocks were diluted into PBS containing 0.1 mg/mL bovine serum albumin (ThermoFisher Scientific, Waltham, MA, USA) to improve stability. Velocity vs. substrate concentration data were fit to the Henri–Michaelis–Menten equation (Equation (1)) [37]:

*v* = V_max_[S]/(*K*_m_ + [S]) (1)

using Kaleidagraph version 4.5 (Synergy Software, Reading, PA, USA).

Experimental evidence so far suggests that BATSIs bind to β-lactamases according to the following mechanism (Equation (2)) [38]:

E + I ⇌ E:I ⇌ E-I*(2)

where E:I represents a reversible binding complex and E-I* represents the covalent, reversible complex mimicking the transition state of either the acylation or deacylation reaction of a β-lactam substrate.

The concentration of inhibitor required to reduce FOX-4’s activity by 50% (IC_50_) was determined using NCF (140 uM) as the reporter substrate, and represents the concentration of inhibitor that gives 50% of the initial velocity. The parameter was estimated from the y-intercept of the linear plot of reciprocal initial velocity versus inhibitor concentration (Equation (3)) [34]:

1/v_0_ = *K*_m_/(V_max_[S]) × (1 + [I]/IC_50_) + 1/V_max_. (3)

BATSIs were pre-incubated with FOX-4 for 5 min before addition of NCF. The IC_50_ values were adjusted for the use of NCF as previously described [29].

X-ray crystallography. Diffraction quality crystals for FOX-4 were grown by sitting drop vapor diffusion by mixing 1 µL of protein (15 mg/mL in 50 mM Tris:HCl, pH 7.5) with 1 µL of reservoir solution and equilibrating the samples against the corresponding reservoir solution. The reservoir solution contained 0.05 M zinc acetate and 20% PEG 3350. Crystals with dimensions 0.1 × 0.1 × 0.2 mm^3^ were mounted in cryo-loops directly from the crystallization droplet and flash cooled in liquid nitrogen. Prior to freezing, 20% glycerol was added to the drop as a cryo-protectant. Both soaking in the drop and co-crystallization were used to obtain structures of the complexes with inhibitors. Diffraction data were recorded on a Rayonix 225 HE CCD detector (Ryonyx, L.L.C., Evanston, IL, USA) with 0.979 Å wavelength radiation on the LRL-CAT beamline (Advanced Photon Source, Argonne, IL, USA). Intensities were integrated using the HKL2000 program and reduced to amplitudes using the SCALEPACK2MTZ program (see Table 1 for statistics) [39,40]. All structures were solved using molecular replacement with PHASER [41] and a PDB 1ZKJ structure as a starting model. Final model building and refinement was performed with the programs COOT, REFMAC, and PHENIX, respectively [42,43,44]. The quality of the final structures was verified with composite omit maps, and stereochemistry was checked with the program MOLPROBITY [45]. LSQKAB and SSM algorithms were used for structural superpositions [46,47]. All other calculations were conducted using the CCP4 program suite [39]. Structures were deposited in the Protein Data Bank (PDB) as 5CHJ (FOX-4/SM23) and 5CHM (FOX-4/LP06).

Structure Visualization. Protein and ligand superpositions were made using the *align* function in PyMol (Schrödinger) using all atoms of the complexes being compared. Omit maps revealing high quality density consistent with the inhibitors were calculated as described below. The final coordinates (5CHM and 5CHJ) were modified with PDBSET (CCP4) by removal of the ligand atoms, uniform adjustment of all B-factors to 20, and adjustment of atomic positions of all remaining atoms by application of random shifts of up to 0.2 Å. The resulting files were subjected to standard refinement with Refmac5 (CCP4) for 40 cycles with tight geometric restraints. Difference maps (2mFo-DFc and mFo-DFc) were calculated with FFT (CCP4) and contoured with MAPMASK (CCP4) around the polypeptide coordinates.

## 3. Results

Combining CAZ with a BATSI lowers the CAZ MICs for E. coli carrying *bla*_FOX-4_. To assess the potency of the inhibitors in whole cells, broth microdilution MIC testing was performed (Table 2). When chiral *m*-carboxyphenyl cephalothin (CEF) analog (SM23) was paired with the β-lactam CAZ, the MIC for CAZ decreased from ≥128 to 4 µg/mL for *E. coli* TG1 pBGS18^−^ *bla*_FOX-4_ (to 32 µg/mL for *E. coli* 42015 *bla*_FOX-4_). The extra carbon linker present on the cephalothin (CEF*) analog (EC04) resulted in decreased potency of CAZ. The cefotaxime analog (CTX-like) compound decreases the MIC values from 128 to 16 µg/mL for *E. coli* TG1 pBGS18^−^ *bla*_FOX-4_.

The extra carboxyl group present on the CAZ analog (LP06) may be beneficial for cell penetration, as the MIC values decreased significantly from 128 to 1 µg/mL for *E. coli* TG1 pBGS18^−^ *bla*_FOX-4_. In addition, the CAZ–LP06 MIC values for the clinical *E*. *coli* 42015 *bla*_FOX-4_ strain were the lowest (16 µg/mL) of the group of CAZ–BATSI combinations tested.

BATSIs are potent inhibitors of the FOX-4 cephamycinase. FOX-4 was probed with four BATSIs (Figure 1) to determine IC_50_ values (Table 3). BATSIs bind tightly to FOX-4 and show the same trend in binding affinity as for AmpC (*E. coli*) and PDC-3 (*Pseudomonas-*derived cephalosporinase). The tightest FOX-4-binding compound, SM23 (CEF analog), has a IC_50_ that is 3-fold lower than LP06 (CAZ analog), running counter to the MIC result: LP06 reduces the CAZ MIC two logs more than SM23. In agreement with the MIC data, however, the EC04 (CEF* analog) binds an order of magnitude less well than SM23 (CEF analog). LP06 (CAZ analog) mimics the CTX-like BATSI, and binds 2.5-fold more tightly, suggesting that the extended oxime side chain encourages binding.

Complex of FOX-4 with SM23. Both crystal soaking and co-crystallization were employed to reveal the structure of the complex. Initially, a stock solution of SM23 (CEF analog) in DMSO was added directly to the crystallization drops containing crystals. However, DMSO damaged the crystals in a time- and concentration-specific manner. The best result was obtained by soaking crystals for 15 min in the 4 µL drop after addition of 0.1 µL 250 mM SM23 (CEF analog) in DMSO. The crystal diffracted to 1.2 Å, with only partial inhibitor occupancy. Alternatively, FOX-4 was incubated with 2 mM SM23 (CEF analog) and then crystallized. Both structures are virtually identical, but co-crystallization achieves full occupancy and this structure was deposited in the PDB (5CHJ).

SM23 (CEF analog) binding results in larger unit cell (Table 1). While apo enzyme crystals contain one FOX-4 chain per asymmetric unit [33], SM23 (CEF analog) complex crystals contain two chains per asymmetric unit with an RMSD of 0.27 (0.52) Å between Cα (all) atoms. The largest difference occurs in the loop near T213, just out of contact with the inhibitor.

**Figure 2:**
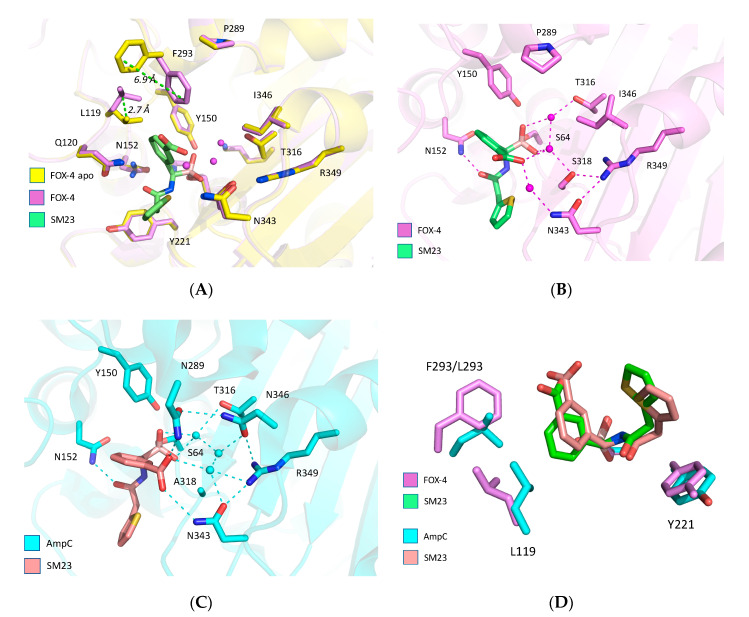

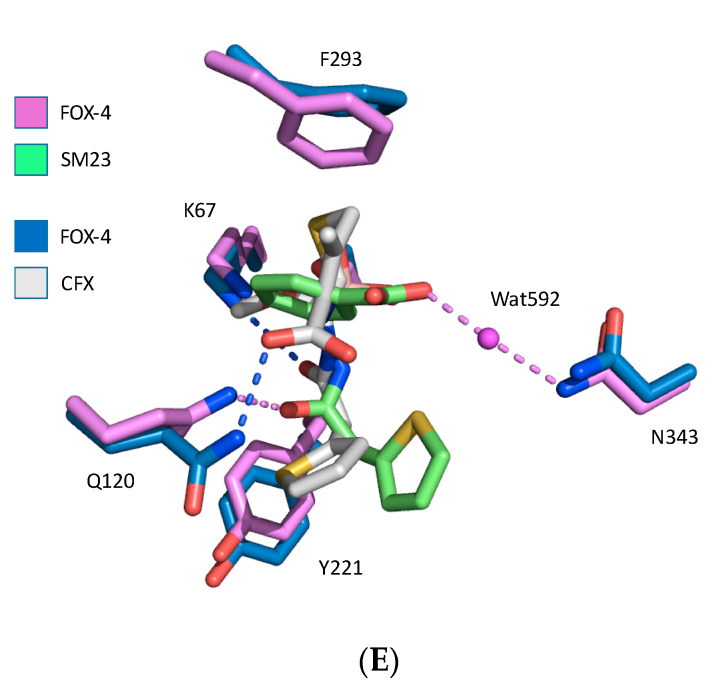
Structures of FOX-4, apo and bound to CEF BATSI (SM23). (**A**) Conformational changes in only F293 and L119 side chains occur upon binding to ligand. The thiophene ring of SM23 does not interact appreciably with Y221. (**B**) Water molecules bridge interactions between the protein (T316, S318, and N343) and borate/carboxylate of the ligand. (**C**) *E. coli* AmpC bound to SM23 (PDB: 1MXO) shows an extensive hydrogen bonded network around N346 and N289. (**D**) SM23 binding modes in FOX-4 (pink) and AmpC (blue) show minor repositioning due to changes in F293/L119 rotation. (**E**) Overlay of FOX-4 structures with SM23 (violet and green) and cefoxitin (blue and white, PDB: 5CGX). Dashed lines indicate hydrogen bonds. Water 592 appears in the SM23 structure only. Carboxylate of SM23 binds in canonical pocket (N343), whereas carboxylate of cefoxitin binds to Q120.

The boron covalently binds to S64 (Figure 2A). The two boronate oxygens adopt positions consistent with all prior BATSI structures in class C enzymes; one oxygen points into the oxyanion hole and the other takes the presumed position of the deacylating water [21]. The carboxyphenyl ring of SM23 (CEF analog) binds in the canonical binding site of cephalosporins, via hydrogen bonds between the carboxylate, S318, and N343 that are mediated by water molecules (Figure 2B). The BATSI carboxyphenyl ring appears to form parallel-displaced π-stacking interactions with the side chain of F293, a residue that is unique to the FOX family. We note that the repositioning of L119 seems to be in response to the large conformational change of F293 between the apo and liganded forms. Additionally, the thiophene ring extends out of the active site and does not make significant contacts; instead, it veers away from the Y221 side chain. By comparison, the *E. coli* AmpC-SM23 (CEF analog) structure (1MXO) has the thiophene ring 1.9 Å closer to Y221 (Figure 2D). The thiophene conformations of SM23 (CEF analog) are unlike the cognate structures of CEF and cefoxitin in complex with the *E*. *coli* AmpC, where parallel π-stacking interactions are evident.

Overall, the SM23 (CEF analog) adopts a similar shape in both FOX-4 and the *E*. *coli* AmpC, with the positioning differences apparently linked to the side chain conformation of L119 and the steric bulk of F293 versus L293 (Figure 2C,D). Notably, the amide carbonyls in both structures point toward N152 in spite of these side chain differences, suggesting a common requirement for binding.

Cefoxitin contains the same R1 side chain as SM23 (CEF analog), and FOX-4 uses the same active site elements to bind both molecules. However, a structure of FOX-4 Y150F (deacylation-impaired) acylated with cefoxitin shows that the dihydrothiazine ring of cefoxitin is rotated 100° relative to the carboxyphenyl ring of SM23 (CEF analog), positioning the C_4_-carboxylate within hydrogen-bonding distance of Q120 (Figure 2E). The ring orientation of cefoxitin may be associated with formation of a product-like conformation that speeds its deacylation. Since the carboxylate of the carboxyphenyl ring of SM23 (CEF analog) binds in the canonical region consisting of S318, N343, and R349 in FOX-4, this interaction may be the most significant one for increasing binding affinity. All crystal structures of bound BATSIs with a carboxyphenyl ring (i.e., SM23 (CEF analog) and EC04 (CEF* analog)) show the same conformation of this group, though the precise residues involved vary across class C β-lactamases [48,49]. It may be that the direct hydrogen bond to Q120 seen in the FOX-4 Y150F/cefoxitin (PDB: 5CGX) and *E*. *coli* AmpC N152G/cefoxitin (PDB: 4KEN) structures is either conformationally unfavorable for the BATSI carboxylate, or that the canonical site is that much more appealing.

Complex of FOX-4 with LP06 (CAZ analog). Both crystal soaking and co-crystallization were employed to reveal the structure of the complex of FOX-4 with LP06 (CAZ analog). LP06 has the R1 side chain of CAZ and aztreonam. Firstly, LP06 (CAZ analog) solution in DMSO was added to the crystallization drop to bring the inhibitor concentration to 9 mM, and the crystal was frozen after 2 h. The electron density map at a resolution of 1.9 Å shows the LP06 (CAZ analog) molecule covalently attached to S64, but the oxyimino side chain, past the imine nitrogen, is missing. This structure was deposited in the PDB (5CHM).

For co-crystallization, protein was incubated with 5 mM LP06 (CAZ analog) and then crystallized. The structure was solved at a resolution of 1.7 Å. In contrast to the SM23 (CEF analog) complex in which inhibitor binding caused change in the unit cell parameters, the LP06 (CAZ analog) complex has the same unit cell as apo FOX-4. Electron density in the co-crystallized structure was more difficult to interpret: just like the soaked complex, the oxime side chain is missing, and the aminothiazole ring has two poses that differ from the soaked complex. This structure was not considered further.

**Figure 3:**
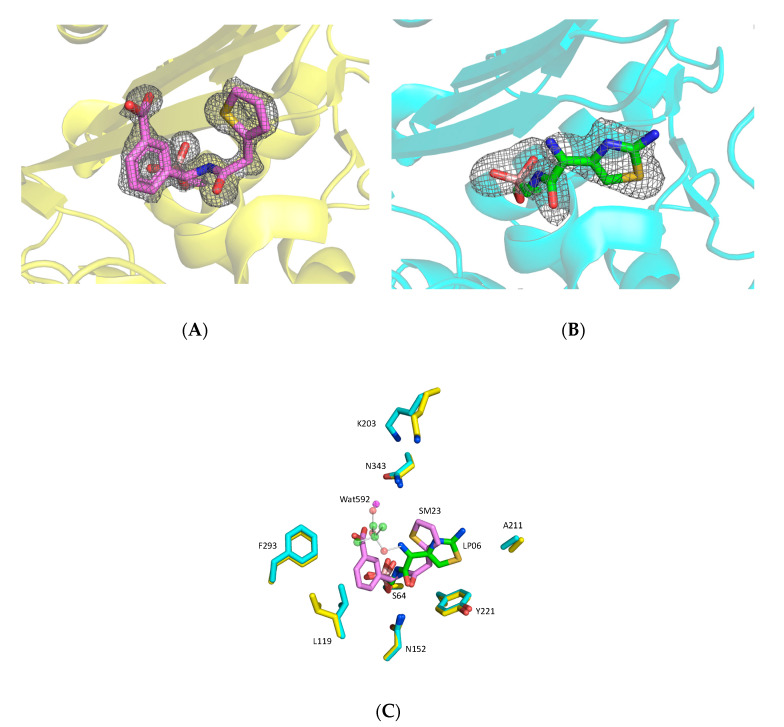
Comparison of BATSIs binding to FOX-4. (**A**,**B**) Omit maps of the ligand electron density were contoured at 3.0 R.M.S.D. for SM23 (CEF analog) and LP06 (CAZ analog) (A and B, respectively). (**C**) Overlay of the FOX-4/SM23 complex (yellow/violet) and FOX-4/LP06 complex (cyan/green) structures. The BATSIs adopt very different conformations. The aminothiazole ring of LP06 forms edge-to-face π-stacking interactions with Y221, while the thiophene ring of SM23 points away. The amide carbonyls both form hydrogen bonds with N152. The missing electron density for LP06 is modeled by the structure from *E. coli* AmpC (PDB: 1IEM). L119 moves away in the SM23 structure.

The visible portions of LP06 (CAZ analog) bound to FOX-4 (PDB: 5CHM) strongly resemble LP06 bound to *E*. *coli* AmpC (PDB: 1IEM), and are consistent with CAZ in the acyl-enzyme of *E*. *coli* AmpC (PDB: 1IEL) (Figure 3). The side chain of LP06 (CAZ analog) has been modeled (ball and stick), for reference from the *E*. *coli* AmpC-LP06 structure. In these three structures, in contrast to SM23 (CEF analog), the R1 ring of LP06 (CAZ analog) (aminothiazole) makes edge-to-face π-stacking interactions with the side chain of Y221. The somewhat more extended chain still retains the amide carbonyl hydrogen bond to N152. The bulky oxime group, which is missing in the FOX-4 structure, is well resolved in the *E*. *coli* AmpC structures with the carboxylate oriented toward its canonical carboxylate binding pocket (N343, R349). The Ω loop may participate in binding of this carboxylate through an extended hydrogen-bonded network including K203.

## 4. Discussion

BATSIs are a class of promising transition-state analog inhibitors that have advanced toward clinical use. Key to their continued development will be better structural analysis showing their interactions with a wide range of targets. BATSI-bound crystal structures for class A β-lactamases are well established, but class C enzymes do not yet enjoy the same attention—most structures are with a single enzyme, *E*. *coli* AmpC. The BATSI-bound FOX-4 structures presented here, in addition to the class C ADC-7 and PDC-3 structures, add a significant new understanding to this group of enzymes.

Lefurgy et al. determined the crystal structure of apo FOX-4 and the acyl-enzyme of cefoxitin with the Y150F variant [33]. The cefoxitin-bound structures show only a few differences in enzyme conformation with the BATSI structures. When cefoxitin is bound, L119 adopts the canonical conformation, the F293 side chain descends 4.0 Å further toward the active site, and the N152 side chain is rotated away from the active site. The latter difference seems to be the result of a repositioning of the cefoxitin amide carbonyl by ~90°, relative to the BATSIs, placing it in hydrogen-bonding distance to K67 and out of range of its usual binding partner, N152. These differences are likely explained by the steric hindrance between cefoxitin’s 7α-methoxy group and N152 that results in a rearrangement not seen in the BATSI conformation.

The R1 side chain rings adopt different conformations in FOX-4. In cefoxitin and LP06 (CAZ analog), the ring is perpendicular with Y221 and makes side-to-face π-stacking interactions; in SM23 (CEF analog), it moves out of range. It appears that the Y221 interaction is not predictive of binding affinity overall.

**Figure 4:**
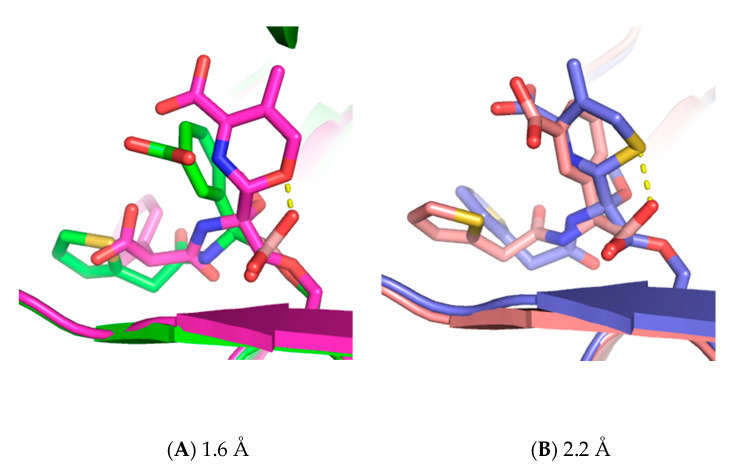
(**A**) Overlay of *E. coli* AmpC bound to SM23 (CEF analog) (green) or moxalactam (purple). (**B**) Overlay of FOX-4 bound to SM23 (pink) or cefoxitin (blue). O1 is placed in the oxyanion hole. O2 presumptive position of deacylating water molecule in the transition state. The dashed line indicates the distance between the dihydrothiazine ring and O2. The longer distance for FOX-4 suggests that the attacking water is more easily accommodated in this active site, explaining the rapid turnover of cephamycins.

Transition-state analogues such as SM23 (CEF analog) can give insight into the mechanism of β-lactamase. As seen in the AmpC-CAZ/BATSI structures (PDB: 1IEL and 1IEM), the geometric constraints on deacylation often require a conformational change by the acylating substrate that is not always readily accommodated. In those structures, the position of the boronic oxygen that represented the presumed deacylation water molecule was observed in the overlay to be too close to the ring nitrogen of dihydrothiazine, suggesting that the substrate itself was occluding the water from its required deacylation trajectory. So too, in the AmpC-moxalactam structure (PDB: 1FCO), we see that the ring comes within 1.6 Å of the boronic oxygen, suggesting that this substrate also prevents water from binding in a deacylation-competent position (Figure 4A). Making the same comparison in FOX-4-cefoxitin/SM23 (CEF analog) structures (PDB: 5CGX and 5CHJ), we see that the acyl-cefoxitin is able to adopt a ring conformation that places the sulfur 2.2 Å away from the boronic oxygen, enough room (presumably) for a water molecule to make an attack trajectory (Figure 4B). The repositioning of the ring into this conformation is possible because of a key bond rotation about C7-C8 that is possible in the FOX-4 active site, but which appears to be sterically occluded in AmpC. The structural differences here are subtle and will require further analysis to fully understand, but may include a slight widening of the active site due to replacement at position 153 of serine (AmpC) with proline (FOX-4).

The enzyme affinity of the BATSIs is not necessarily a good predictor of their in vivo activity. The IC_50_ values for SM23 (CEF analog) suggested that it would be more potent than LP06 (CAZ analog) (0.032 μM vs. 0.11 μM), yet MIC values revealed that LP06 was in fact more potent than SM23 (1 vs. 4 μg/mL for the laboratory strain and and 16 vs. 32 μg/mL for the clinical strain)—perhaps owing to increased penetration by LP06. The overall higher resistance of the clinical strain may be linked to other resistance mechanisms, such as mutations in outer membrane proteins or the presence of drug efflux pumps. The slightly reduced overall affinity of BATSIs for FOX-4, compared to *E*. *coli* AmpC and PDC-3, may be rationalized by considering the replacements N346I and N289P, resulting in a much sparser hydrogen-bonding network involving the *m*-carboxyphenyl group (Figure 2C). The same replacements may account for the disordered oxime side chain of LP06 (CAZ analog), which binds in the same location. It is notable that the cefotaxime-like BATSI shows a slightly lower affinity than LP06 (CAZ analog), suggesting that while the larger oxime side chain of LP06 is disordered, it does contribute somewhat to the overall affinity.

## 5. Conclusions

A significant result of this study is the observation that a BATSI can bind with nanomolar affinity to quite different class C enzyme active sites by adopting different conformations. This echoes a similar observation in class A enzymes for the SM23 (CEF analog) inhibitor (SMS2, [10]). The “flexible” inhibitor that undergoes structural shifts in concert with its target enzyme (i.e., induced fit involving changes in both ligand and protein) may be a paradigm for future inhibitor design. This observation runs counter to what is known about cyclic boronates, which are more conformationally constrained but have a broader spectrum.

## Figures and Tables

**Table 1:** Data collection and refinement statistics for the FOX-4 crystal structures.

	WT	WT
Ligand	SM23 cephalothin (CEF) analog	LP06 ceftazidime (CAZ) analog
PDB entry	**5CHJ**	**5CHM**
**Data Collection**		
Resolution range (Å)	20.0–1.4	20.0–1.9
Wavelength (Å)	0.979	0.979
Space group	P2_1_	P2_1_
Unit cell dimensions (Å)	a = 74.29	a = 54.85
	b = 57.16	b = 57.09
	c = 83.23	c = 56.75
	α = γ = 90°	α = γ = 90°
	β = 91.67°	β = 96.45°
Observed reflections	539,219	94,986
Unique reflections	147,034	27,189
Completeness (%) ^a^	97.8 (96.5)	98.8 (98.0)
*I/σ*	9.9 (2.6)	7.6 (2.8)
Rmerge (I) ^b^	0.067 (0.448)	0.094 (0.422)
**Structure Refinement**		
Rcryst (%) ^c^	0.163	0.170
Rfree (%) ^c^	0.192	0.228
Protein non-hydrogen atoms	5511	2722
Water molecules	1009	237
Average B-factor (Å^2^)	9.5	15.6
**RMS Deviations from Ideal Value**		
Bonds (Å)	0.011	0.007
Angles (°)	1.46	1.09
Torsion angles (°)	13.5	13.5
Overall coordinate error		
(Maximum likelihood)	0.11	0.22
**Ramachandran Statistics (%)**		
(For non-Gly/Pro residues)		
Most favorable	98.1	97.8
Additional allowed	1.9	2.2

^a^ Values in parentheses indicate statistics for the high-resolution bin. ^b^ R_merge_ = ΣΣ _j_|I_j_(hkl) − <I(hkl)>|/ΣΣ _j_|<I(hkl)>|, where I_j_ is the intensity measurement for reflection j and <I> is the mean intensity over j reflections. ^c^ R_cryst_/(R_free_) = Σ ||F_o_(hkl) − |F_c_(hkl)||/Σ |F_o_(hkl)|, where F_o_ and F_c_ are observed and calculated structure factors, respectively. In total, 5% of the reflections were excluded from refinement and used to calculate R_free_.

**Table 2:** Broth microdilutions Minimum Inhibitory Concentrations (MIC) results.

Strain	CAZ ^#^ Only	CAZ/SM23 (CEF-Like)	CAZ/EC04 (CEF *-Like)	CAZ/CTX-Like	CAZ/LP06 (CAZ-Like)
*E. coli bla*_FOX-4_	>128	32	64	64	16
*E. coli* TG1 pBGS18-*bla*_FOX-4_	128	4	16	16	1

# Concentrations of ceftazidime (CAZ) are in μg/mL; BATSI is present at a constant concentration of 10 μg/mL; the CLSI breakpoint for CAZ resistance is an MIC ≥ 16 μg/mL. Cephalothin (CEF); cefotaxime (CTX). * An extra carbon linker is present on the R2 side chain.

**Table 3:** IC_50_ values for boronic acid transition-state analog inhibitor (BATSI) compounds.

BATSI	FOX-4 ^1^ IC_50_ (μM)	*E. coli* AmpC ^2^ IC_50_ (μM)	PDC-3 ^3^ IC_50_ (μM)
SM23 (CEF-like)	0.032 ± 0.003	0.001	0.004
EC04 (CEF *-like)	0.42 ± 0.02	n.d.	0.22
CTX-like	0.26 ± 0.02	0.31 ^4^	0.17
LP06 (CAZ-like)	0.11 ± 0.02	0.020 ^4^	0.004

^1^ This study. ^2^ Ref [6,21]. ^3^ Ref [34]. ^4^ No time-dependent inhibition was observed, so these values represent true *K*_i_ values. Cephalothin (CEF); cefotaxime (CTX); ceftazidime (CAZ). * An extra carbon linker is present on the R2 side chain.

## References

[1] Papp-Wallace K.M., Endimiani A., Taracila M.A., Bonomo R.A. (2011). Carbapenems: Past, Present, and Future. Antimicrob. Agents Chemother..

[2] Drawz S.M., Bonomo R.A. (2010). Three Decades of beta-Lactamase Inhibitors. Clin. Microbiol. Rev..

[3] Krajnc A., Lang P.A., Panduwawala T.D., Brem J., Schofield C.J. (2019). Will morphing boron-based inhibitors beat the β-lactamases?. Curr. Opin. Chem. Biol..

[4] Papp-Wallace K.M., Bonomo R.A. (2016). New β-lactamase Inhibitors in the Clinic. Infect. Dis. Clin. N. Am..

[5] Caselli E., Powers R.A., Blasczcak L.C., Wu C.Y., Prati F., Shoichet B.K. (2001). Energetic, structural, and antimicrobial analyses of beta-lactam side chain recognition by beta-lactamases. Chem. Biol..

[6] Morandi F., Caselli E., Morandi S., Focia P.J., Blázquez J., Shoichet B.K., Prati F. (2003). Nanomolar Inhibitors of AmpC β-lactamase. J. Am. Chem. Soc..

[7] Drawz S.M., Papp-Wallace K.M., Bonomo R.A. (2014). New beta-Lactamase Inhibitors: A Therapeutic Renaissance in an MDR World. Antimicrob. Agents Chemother..

[8] Werner J.P., Mitchell J.M., Taracila M.A., Bonomo R.A., Powers R.A. (2017). Exploring the potential of boronic acids as inhibitors of OXA-24/40 β-lactamase. Protein Sci..

[9] Venturelli A., Tondi D., Cancian L., Morandi F., Cannazza G., Segatore B., Prati F., Amicosante G., Shoichet B.K., Costi M.P. (2007). Optimizing Cell Permeation of an Antibiotic Resistance Inhibitor for Improved Efficacy. J. Med. Chem..

[10] Tondi D., Venturelli A., Bonnet R., Pozzi C., Shoichet B.K., Costi M.P. (2014). Targeting Class A and C Serine β-lactamases with a Broad-Spectrum Boronic Acid Derivative. J. Med. Chem..

[11] Cendron L., Quotadamo A., Maso L., Bellio P., Montanari M., Celenza G., Venturelli A., Costi M.P., Tondi D. (2019). X-ray-crystallography deciphers the activity of broad-spectrum boronic acid β-lactamase inhibitors. ACS Med. Chem. Lett..

[12] Brem J., Cain R., Cahill S., McDonough M.A., Clifton I.J., Jiménez-Castellanos J.-C., Avison M.B., Spencer J., Fishwick C.W.G., Schofield C.J. (2016). Structural basis of metallo-β-lactamase, serine-β-lactamase and penicillin-binding protein inhibition by cyclic boronates. Nat. Commun..

[13] Cahill S.T., Cain R., Wang D.Y., Lohans C.T., Wareham D.W., Oswin H.P., Mohammed J., Spencer J., Fishwick C.W.G., McDonough M.A. (2017). Cyclic Boronates Inhibit All Classes of β-lactamases. Antimicrob. Agents Chemother..

[14] Cahill S.T., Tyrrell J.M., Navratilova I.H., Calvopiña K., Robinson S.W., Lohans C.T., McDonough M.A., Cain R., Fishwick C.W.G., Avison M.B. (2019). Studies on the inhibition of AmpC and other β-lactamases by cyclic boronates. Biochim. Biophys. Acta Gen. Subj..

[15] Hecker S.J., Reddy K.R., Totrov M., Hirst G.C., Lomovskaya O., Griffith D.C., King P., Tsivkovski R., Sun D., Sabet M. (2015). Discovery of a Cyclic Boronic Acid β-lactamase Inhibitor (RPX7009) with Utility vs. Class A Serine Carbapenemases. J. Med. Chem..

[16] Zhanel G.G., Lawrence C.K., Adam H., Schweizer F., Zelenitsky S., Zhanel M., Lagacé-Wiens P.R.S., Walkty A., Denisuik A., Golden A. (2018). Imipenem-Relebactam and Meropenem-Vaborbactam: Two Novel Carbapenem-β-lactamase Inhibitor Combinations. Drugs.

[17] Bush K. (2018). Game Changers: New β-lactamase Inhibitor Combinations Targeting Antibiotic Resistance in Gram-Negative Bacteria. ACS Infect. Dis..

[18] Hecker S.J., Reddy K.R., Lomovskaya O., Griffith D.C., Rubio-Aparicio D., Nelson K., Tsivkovski R., Sun D., Sabet M., Tarazi Z. (2020). Discovery of Cyclic Boronic Acid QPX7728, an Ultra-broad-spectrum Inhibitor of Serine and Metallo Beta-lactamases. J. Med. Chem..

[19] Tsivkovski R., Totrov M., Lomovskaya O. (2020). Biochemical Characterization of QPX7728, a New Ultra-Broad-Spectrum Beta-lactamase Inhibitor of Serine and Metallo-Beta-Lactamases. Antimicrob. Agents Chemother..

[20] Krajnc A., Brem J., Hinchliffe P., Calvopiña K., Panduwawala T.D., Lang P.A., Kamps J.J.A.G., Tyrrell J.M., Widlake E., Saward B.G. (2019). Bicyclic Boronate VNRX-5133 Inhibits Metallo- and Serine-β-lactamases. J. Med. Chem..

[21] Powers R.A., Caselli E., Focia P.J., Prati F., Shoichet B.K. (2001). Structures of ceftazidime and its transition-state analogue in complex with AmpC beta-lactamase: Implications for resistance mutations and inhibitor design. Biochemistry.

[22] Chen Y., Shoichet B., Bonnet R. (2005). Structure, function, and inhibition along the reaction coordinate of CTX-M beta-lactamases. J. Am. Chem. Soc..

[23] Ness S., Martin R., Kindler A.M., Paetzel M., Gold M., Jensen S.E., Jones J.B., Strynadka N.C. (2000). Structure-based design guides the improved efficacy of deacylation transition state analogue inhibitors of TEM-1 beta-Lactamase. Biochemistry.

[24] Nguyen N.Q., Krishnan N.P., Rojas L.J., Prati F., Caselli E., Romagnoli C., Bonomo R.A., van den Akker F. (2016). Crystal Structures of KPC-2 and SHV-1 β-lactamases in Complex with the Boronic Acid Transition State Analog S02030. Antimicrob. Agents Chemother..

[25] Weston G.S., Blázquez J., Baquero F., Shoichet B.K. (1998). Structure-based enhancement of boronic acid-based inhibitors of AmpC beta-lactamase. J. Med. Chem..

[26] Tondi D., Powers R.A., Caselli E., Negri M.C., Blázquez J., Costi M.P., Shoichet B.K. (2001). Structure-based design and in-parallel synthesis of inhibitors of AmpC beta-lactamase. Chem. Biol..

[27] Chen Y., Minasov G., Roth T.A., Prati F., Shoichet B.K. (2006). The deacylation mechanism of AmpC beta-lactamase at ultrahigh resolution. J. Am. Chem. Soc..

[28] Caselli E., Romagnoli C., Powers R.A., Taracila M.A., Bouza A.A., Swanson H.C., Smolen K.A., Fini F., Wallar B.J., Bonomo R.A. (2018). Inhibition of Acinetobacter-Derived Cephalosporinase: Exploring the Carboxylate Recognition Site Using Novel β-lactamase Inhibitors. ACS Infect. Dis..

[29] Bouza A.A., Swanson H.C., Smolen K.A., VanDine A.L., Taracila M.A., Romagnoli C., Caselli E., Prati F., Bonomo R.A., Powers R.A. (2018). Structure-Based Analysis of Boronic Acids as Inhibitors of Acinetobacter-Derived Cephalosporinase-7, a Unique Class C β-lactamase. ACS Infect. Dis..

[30] Cejas D., Fernández Canigia L., Quinteros M., Giovanakis M., Vay C., Lascialandare S., Mutti D., Pagniez G., Almuzara M., Gutkind G. (2012). Plasmid-Encoded AmpC (pAmpC) in Enterobacteriaceae: Epidemiology of microorganisms and resistance markers. Rev. Argent. Microbiol..

[31] Jacoby G.A. (2009). AmpC-Lactamases. Clin. Microbiol. Rev..

[32] Bou G., Oliver A., Ojeda M., Monzón C., Martínez-Beltrán J. (2000). Molecular characterization of FOX-4, a new AmpC-type plasmid-mediated beta-lactamase from an Escherichia coli strain isolated in Spain. Antimicrob. Agents Chemother..

[33] Lefurgy S.T., Malashkevich V.N., Aguilan J.T., Nieves E., Mundorff E.C., Biju B., Noel M.A., Toro R., Baiwir D., Papp-Wallace K.M. (2016). Analysis of the Structure and Function of FOX-4 Cephamycinase. Antimicrob. Agents Chemother..

[34] Drawz S.M., Taracila M., Caselli E., Prati F., Bonomo R.A. (2011). Exploring sequence requirements for C3/C4 carboxylate recognition in the Pseudomonas aeruginosa cephalosporinase: Insights into plasticity of the AmpC β-lactamase: C3/C4 Carboxylate Recognition in P. aeruginosa AmpC. Protein Sci..

[35] Mallo S., Pérez-Llarena F.J., Kerff F., Soares N.C., Galleni M., Bou G. (2010). A tripeptide deletion in the R2 loop of the class C beta-lactamase enzyme FOX-4 impairs cefoxitin hydrolysis and slightly increases susceptibility to beta-lactamase inhibitors. J. Antimicrob. Chemother..

[36] Patel J.B., Clinical and Laboratory Standards Institute (2017). Performance Standards for Antimicrobial Susceptibility Testing.

[37] Michaelis L., Menten M. (1913). Die Kinetik der Invertinwirkung. Biochemische Zeitschrift.

[38] Crompton I.E., Cuthbert B.K., Lowe G., Waley S.G. (1988). Beta-lactamase inhibitors. The inhibition of serine beta-lactamases by specific boronic acids. Biochem. J..

[39] Winn M.D., Ballard C.C., Cowtan K.D., Dodson E.J., Emsley P., Evans P.R., Keegan R.M., Krissinel E.B., Leslie A.G.W., McCoy A. (2011). Overview of the CCP 4 suite and current developments. Acta Crystallogr. D Biol. Crystallogr..

[40] Otwinowski Z., Minor W. (1997). Processing of X-ray diffraction data collected in oscillation mode. Methods in Enzymology.

[41] McCoy A.J., Grosse-Kunstleve R.W., Adams P.D., Winn M.D., Storoni L.C., Read R.J. (2007). Phaser crystallographic software. J. Appl. Crystallogr..

[42] Adams P.D., Grosse-Kunstleve R.W., Hung L.W., Ioerger T.R., McCoy A.J., Moriarty N.W., Read R.J., Sacchettini J.C., Sauter N.K., Terwilliger T.C. (2002). PHENIX: Building new software for automated crystallographic structure determination. Acta Crystallogr. D Biol. Crystallogr..

[43] Emsley P., Cowtan K. (2004). Coot: Model-building tools for molecular graphics. Acta Crystallogr. D Biol. Crystallogr..

[44] Murshudov G.N., Vagin A.A., Dodson E.J. (1997). Refinement of macromolecular structures by the maximum-likelihood method. Acta Crystallogr. D Biol. Crystallogr..

[45] Chen V.B., Arendall W.B., Headd J.J., Keedy D.A., Immormino R.M., Kapral G.J., Murray L.W., Richardson J.S., Richardson D.C. (2010). MolProbity: All-atom structure validation for macromolecular crystallography. Acta Crystallogr. D Biol. Crystallogr..

[46] Kabsch W. (1976). A solution for the best rotation to relate two sets of vectors. Acta Crystallogr. A.

[47] Krissinel E., Henrick K. (2004). Secondary-structure matching (SSM), a new tool for fast protein structure alignment in three dimensions. Acta Crystallogr. D Biol. Crystallogr..

[48] Beadle B.M., Trehan I., Focia P.J., Shoichet B.K. (2002). Structural milestones in the reaction pathway of an amide hydrolase: Substrate, acyl, and product complexes of cephalothin with AmpC beta-lactamase. Structure.

[49] Beadle B.M., Shoichet B.K. (2002). Structural basis for imipenem inhibition of class C beta-lactamases. Antimicrob. Agents Chemother..

